# Application of a time-fractal fractional derivative with a power-law kernel to the Burke-Shaw system based on Newton's interpolation polynomials

**DOI:** 10.1016/j.mex.2023.102510

**Published:** 2023-12-14

**Authors:** Najat Almutairi, Sayed Saber

**Affiliations:** a Department of Mathematics, College of Science, Qassim University, Buraidah, Saudi Arabia; b Department of Mathematics, Faculty of Science, Al-Baha University, Saudi Arabia; c Department of Mathematics and Computer Science, Faculty of Science, Beni-Suef University, Egypt

**Keywords:** Fractal-fractional Caputo Method, Fractional derivatives, Nonlinear equations, Simulation, Numerical results, Iterative method, Time varying control system, Lyapunov functions

## Abstract

This paper proposes some updated and improved numerical schemes based on Newton's interpolation polynomial. A Burke-Shaw system of the time-fractal fractional derivative with a power-law kernel is presented as well as some illustrative examples. To solve the model system, the fractal-fractional derivative operator is used. Under Caputo's fractal-fractional operator, fixed point theory proves Burke-Shaw's existence and uniqueness. Additionally, we have calculated the Lyapunov exponent (LE) of the proposed system. This method is illustrated with a numerical example to demonstrate the applicability and efficiency of the suggested method. To analyze this system numerically, we use a fractal- fractional numerical scheme with a power-law kernel to analyze the variable order fractal- fractional system. Furthermore, the Atangana-Seda numerical scheme, based on Newton polynomials, has been used to solve this problem. This novel method is illustrated with numerical examples. Simulated and analytical results agree. We contribute to real-world mathematical applications. Finally, we applied a numerical successive approximation method to solve the fractional model.

• The purpose of this section is to define a mathematical model to study the dynamic behavior of the Burke-Shaw system.
• With the Danca algorithm [1,2] and Adams-Bashforth-Moulton numerical scheme, we compute the Lyapunov exponent of the system, which is useful for studying Dissipativity.
• In a generalized numerical method, we simulate the solutions of the system using the time-fractal fractional derivative of Atangana-Seda.

Specifications Table

**Table:** 

Subject area	Mathematics and Statistics
More specific subject area	Biomathematics
Method name:	Fractal-fractional Caputo Method
Name and reference of original method:	Atangana A, Araz SI (2021) New numerical Scheme with Newton polynomial: theory, methods, and applications. Academic, Cambridge. 978-0323854481.
Resource availability:	This method has been developed in MATLAB

## Method details

The Lorenz attractor was named after Edward Norton Lorenz, who derived it from the simplified equations of convection rolls arising in the atmosphere equations in 1963. Burke and Shaw derived the Burke-Shaw system from the Lorenz system [3]. This system has a similar algebraic structure to the Lorenz system but is topologically nonequivalent to the generalized Lorenz-type system and can be expressed as follows:

$$\frac{dx\left(t\right)}{dt}=\;-a\left(x\left(t\right)+\;y\left(t\right)\right),$$

$$\frac{dy\left(t\right)}{dt}=\;-k\;x\left(t\right)\;z\left(t\right)\;-\;y\left(t\right),$$

$$\frac{dz\left(t\right)}{dt}=\;g\;x\left(t\right)\;y\left(t\right)\;+\;d,$$

where x,y, and z state variables and a,k,g,d are real constants. Its fractional-order version can be expressed as [4]:

$$D_{0,t}^{\alpha }x\left(t\right)=\;-a\left(x\left(t\right)+\;y\left(t\right)\right),$$

$$D_{0,t}^{\alpha }y\left(t\right)=\;-k\;x\left(t\right)\;z\left(t\right)\;-\;y\left(t\right),$$

$$D_{0,t}^{\alpha }z\left(t\right)=\;g\;x\left(t\right)\;y\left(t\right)\;+\;d,$$

where α is the derivative order, which could be arbitrary real numbers. Chaos control is performed by a simple linear controller, and a numerical simulation of the control is provided. In addition, Chen, and Lee [5] introduced a novel chaotic system capable of generating dual-role chaos attractors when investigating rigid body motion anti-chaos control. Richter [6] studied the stability and chaos control of Newton-Leipnik systems [7], [8], [9] using static nonlinear feedback laws based on Lyapunov functions. In [9], Long-Jye Sheu et al., investigated the dynamics of the Newton-Leipnik system with fractional order and was studied numerically. Fractional calculus (FC) allows integration and differentiation of operators in fractional order. Samko proposed a fascinating extension of constant-order FC in [10], [11], [12], [13], [14], [15], [16], [17], [18], [19]. Solis-Perez, et al. [20], introduced fractional operators that consider order as a function of time, space, or other variables. Variable-order fractional differential equations cannot be solved exactly, so developing numerical schemes for solving these equations is crucial. For fractional differential equations, the Adams-Bashforth method is highly effective - see [21,2,22]. In [20], Solis-Perez, et al. developed a constant-order numerical scheme that combines fractional calculus and Lagrange polynomials. Using this method, they generalized the numerical schemes for simulating variable-order fractional differential operators with power-law, exponential-law, and Mittag-Leffler kernels. See also [23], [24], [25], [26], [27], [28], [29], [30], [31], [32], [33], [34], [35], [36], [37], [38], [39], [40]. The aim of this paper is to propose some new improved numerical schemes based on Newton's interpolation polynomial. The purpose of this study is to describe the properties of the Burke- Shaw system in the sense of a time-fractal fractional derivative with a power-law kernel. We examine the numerical aspects of the presented system as well as the existence a uniqueness of the solutions to the presented model. By using the Banach fixed point theorem and Picard iterative method, we look at the stability of fixed points and determine the range at which it can be controlled to be stable. With Lagrange polynomials, a variable-order fractal fractional with a power-law kernel is used to simulate state variables. In summary, the paper makes the following claims:

(a) Demonstrate the effectiveness and uniqueness of the system presented.
(b) Utilizing fractal-fractional derivatives to simulate the solution of the system using a power-law kernel method
(c) Obtaining the Lyapunov exponent for the proposed system.
(d) Simulating the behavior of different state variables and investigating the impact of change the values of some parameters in the model.

## Model formulation

We consider the family Ψ of all increasing functions ψ:[0,∞)→[0,∞) such that

$$\sum_{j=1}^{\infty }\psi^{j}\left(t\right)<\infty ,\;\psi \left(t\right)<\;t,\;\forall t\;>\;0.$$

Definition 1 Let F:X→X and $\varphi :X^{2}\to R_{\geq 0}$, where X is a normed space. Then,

(1) For $\tau_{1},\;\tau_{2}\;\in \;X,\;F$ is ϕ−ψ-contraction if

$$\phi \;\left(\tau_{1},\;\tau_{2}\right)\;d\;\left(F_{\tau_{1}},\;F_{\tau_{2}}\right)\;\leq \;\psi \;\left(d\;(\tau_{1},\;\tau_{2})\right)\;.$$

(2) *F* is ϕ-admissible if

$$\phi \;\left(\tau_{1},\;\tau_{2}\right)\;\geq \;1\;\Rightarrow \;\phi \;\left(F_{\tau_{1}},\;F_{\tau_{2}}\right)\;\geq \;1.$$

Definition 2 [32]. Let a continuous function F:(a,b)→[0,∞) be fractal differentiable of fractal order β. Then, the fractal-fractional derivative of F equipped with the power-law-type kernel of order ω in the sense of Riemann- Liouville is defined by

$${}^{\text{FF}}D_{0,t}^{\alpha ,\beta }y\left(t\right)≔\frac{B\left(\alpha \right)}{1-\alpha }\frac{d}{dt^{\beta }}\int_{0}^{t}{\left(t-\tau \right)}^{n-\alpha -1}\;F\left(\omega \right)d\omega ,\;0<\alpha <1,\;$$

where

$$\frac{dF\left(s\right)}{ds^{\beta }}=\lim_{n\to \infty }\frac{F\left(t\right)-F\left(s\right)}{t^{\beta }-s^{\beta }}$$

is the fractal derivative and n−1<ω,v≤n∈N. Using the fractal-fractional sense of differential and integral operators we get the following Burke- Shaw model:

(1) $$\begin{array}{ccc} {}^{\text{FF}}D_{0,t}^{\alpha ,\beta }x\left(t\right) & = & \;-a\left(x\left(t\right)+\;y\left(t\right)\right),\\ {}^{\text{FF}}D_{0,t}^{\alpha ,\beta }y\left(t\right) & = & \;-k\;x\left(t\right)\;z\left(t\right)\;-\;y\left(t\right),\\ {}^{\text{FF}}D_{0,t}^{\alpha ,\beta }z\left(t\right) & = & \;g\;x(t)y(t)+\;d. \end{array}$$

## Model properties

### Existence and uniqueness

Define the Banach space $U\;=X^{3}$, where X=C(I,R) under the norm

$${∥\mu ∥}_{X}={∥\left(x,y,z\right)∥}_{X}=\max\left\{\left|K\left(t\right)\right|:t\in I\right\},$$

for which |K|≔|x|+|y|+|z|. Let $∥x∥\;\leq \;\lambda_{1},\;∥y∥\;\leq \;\lambda_{2},\;∥z∥\;\leq \;\lambda_{3}$ for some constants $\lambda_{1},\;\lambda_{2},\;\lambda_{3}>0.$

(2) $$\begin{array}{ccc} F_{1}\;\left(x,\;y,\;z\right) & = & -a\left(x\left(t\right)\;+\;y\left(t\right)\right),\\ F_{2}\;\left(x,\;y,\;z\right) & = & -k\;x\left(t\right)\;z\left(t\right)\;-\;y\left(t\right),\\ F_{3}\;\left(x,\;y,\;z\right) & = & g\;x(t)\;y(t)\;+\;d. \end{array}$$

Since the integral is differentiable, we can rewrite the system (1) as follows

$$\begin{array}{ccc} {}^{\text{RL}}D_{0,t}^{\alpha }x\left(t\right) & = & \;\beta t^{\beta -1}F_{1}\;\left(x,\;y,\;z\right),\\ {}^{\text{RL}}D_{0,t}^{\alpha }y\left(t\right) & = & \;\beta t^{\beta -1}F_{2}\;\left(x,\;y,\;z\right),\\ {}^{\text{RL}}D_{0,t}^{\alpha }z\left(t\right) & = & \;\beta t^{\beta -1}F_{3}\;\left(x,\;y,\;z\right). \end{array}$$

Now, when we replace the derivative ${}^{\text{RL}}D$ by ${}^{C}D$*,* applying fractional integral, we get the solution as follows:

(3) $$E\left(t\right)=E\left(0\right)=\frac{\beta }{\Gamma \left(\alpha \right)}\int_{0}^{t}\psi \left(\tau ,E\left(\tau \right)\right){\left(t-\tau \right)}^{\alpha -1}\;\tau^{\beta -1}d\tau ,$$

where

$$E\left(t\right)=\left\{ \begin{array}{c} x\left(t\right)\\ y\left(t\right)\\ z\left(t\right) \end{array}\right.\;E\left(0\right)=\left\{ \begin{array}{c} x\left(0\right)\\ y\left(0\right)\\ z\left(0\right) \end{array}\right.,\;\psi \left(t\right)=\left\{ \begin{array}{c} U\left(x\left(t\right),\;y\left(t\right),\;z\left(t\right)\right)\\ V\left(x\left(t\right),\;y\left(t\right),\;z\left(t\right)\right)\\ W\left(x\left(t\right),\;y\left(t\right),\;z\left(t\right)\right) \end{array}\right.\;.$$

Now, we are going to show existence theorems by proving that *T* is a contraction mapping,

T:X→X

$$E\left(t\right)\to T\left(E\left(t\right)\right)=\frac{\beta }{\Gamma \left(\alpha \right)}\int_{0}^{t}\psi \left(\tau ,E\left(\tau \right)\right){\left(t-\tau \right)}^{\alpha -1}\;\tau^{\beta -1}d\tau ,$$

then there exists a unique fixed point $E_{0}\in X$,

$$\begin{array}{ccc} \|\psi \left(t,E_{1}\left(t\right)\right)-\psi \left(t,E_{2}\left(t\right)\right)∥ & = & ∥E_{1}\left(0\right)+\frac{\beta }{\Gamma \left(\alpha \right)}\int_{0}^{t}{\left(t-\tau \right)}^{\alpha -1}\;\tau^{\beta -1}\psi \left(\tau ,E_{1}\left(\tau \right)\right)d\tau -E_{2}\left(0\right)\\ & & -\frac{\beta }{\Gamma \left(\alpha \right)}\int_{0}^{t}{\left(t-\tau \right)}^{\alpha -1}\;\tau^{\beta -1}\psi \left(\tau ,E_{2}\left(\tau \right)\right)d\tau ∥\leq ∥E_{1}\left(0\right)-E_{2}\left(0\right)∥\\ & & +\frac{\beta }{\Gamma \left(\alpha \right)}∥\int_{0}^{t}{\left(t-\tau \right)}^{\alpha -1}\;\tau^{\beta -1}\left(\psi \left(\tau ,E_{1}\left(\tau \right)\right)-\psi \left(\tau ,E_{1}\left(\tau \right)\right)\right)d\tau ∥\leq ∥E_{1}\left(0\right)-E_{2}\left(0\right)∥\\ & & +\frac{\beta t_{max}^{\alpha +\beta -1}}{\Gamma \left(\alpha \right)}\|\psi \left(\tau ,E_{1}\left(\tau \right)\right)-\psi \left(\tau ,E_{1}\left(\tau \right)\right)∥\leq ∥E_{1}\left(0\right)-E_{2}\left(0\right)∥+\frac{\beta t_{max}^{\alpha +\beta -1}}{\Gamma \left(\alpha \right)}\psi \leq \Phi . \end{array}$$

Let $E_{0}$ is any point in *X,*

$$\begin{array}{ccc} \|E\left(t\right)-E\left(0\right)∥ & = & ∥E\left(0\right)+\frac{\beta }{\Gamma \left(\alpha \right)}\int_{0}^{t}{\left(t-\tau \right)}^{\alpha -1}\;\tau^{\beta -1}\psi \left(\tau ,E\left(\tau \right)\right)d\tau -E\left(0\right)∥\\ & & \leq \frac{\beta }{\Gamma \left(\alpha \right)}∥\int_{0}^{t}{\left(t-\tau \right)}^{\alpha -1}\;\tau^{\beta -1}\psi \left(\tau ,E\left(\tau \right)\right)d\tau ∥\leq \frac{\beta t_{max}^{\alpha +\beta -1}}{\Gamma \left(\alpha \right)}\|\psi \left(\tau ,E\left(\tau \right)\right)∥=\frac{\beta t_{max}^{\alpha +\beta -1}}{\Gamma \left(\alpha \right)}\psi \end{array}$$

so the existence follows. To prove the uniqueness of solution of the given fractal fractional model (1), we use the Lipschitz property of functions U,V,W given by (2). To prove the following results, we need the following conditions:

Theorem 1 *[**33**]. Consider the functions x, y, z, x^∗^, y^∗^, z^∗^ ∈ X. Then, the functions*U,V,W*introduced by (*2*) are satisfied the Lipschitz property with respect to the corresponding components*

if *w*_1_*, w*_2_*, w*_3_ *>* 0, where *w*_1_ = *a, w*_2_ = 1*, w*_3_ = 1*.*

Proof For each $x,\;x^{\prime }\;\in \;X$, we have

$$∥U\left(x\left(t\right),y\left(t\right),\;z\left(t\right),t\right)-U\left(x^{*}\left(t\right),y^{*}\left(t\right),\;z^{*}\left(t\right),t\right)∥\leq w_{1}∥x\left(t\right)-x^{*}\left(t\right)∥.$$

This shows that U is Lipschitz with respect to *x* with the Lipschitz constant $w_{1}>0$.

For each $y,\;y^{\prime }\;\in \;X$, we have

$$∥V\left(x\left(t\right),y\left(t\right),\;z\left(t\right),t\right)-V\left(x^{*}\left(t\right),y^{*}\left(t\right),\;z^{*}\left(t\right),t\right)∥\leq w_{1}∥y\left(t\right)-y^{*}\left(t\right)∥.$$

This shows that *F*_2_ is Lipschitz with respect to *y* with the Lipschitz constant *w*_2_ *>* 0. For each

$z,\;z{}^{\prime }\;\in \;X$, we have

$$∥W\left(x\left(t\right),y\left(t\right),\;z\left(t\right),t\right)-W\left(x^{*}\left(t\right),y^{*}\left(t\right),\;z^{*}\left(t\right),t\right)∥\leq w_{1}∥z\left(t\right)-z^{*}\left(t\right)∥.$$

This shows that *F*_3_ is Lipschitz with respect to *z* with the Lipschitz constant *w*_3_ *>* 0. As a result,

U,V,W are Lipschitzian with respect to the Lipschitz constants $w_{1},\;w_{2},\;w_{3}>0$, respectively.

Theorem 2 *The fractal-fractional model (* 1 *) has a unique solution if*

$$\frac{\beta t^{\beta +\alpha -1}\Gamma \left(\beta \right)}{\Gamma \left(\beta +\alpha \right)}w_{i}<1,\;i\in \left\{1,2,3\right\}.$$

Proof Assume that $x^{*}\left(t\right),\;y^{*}\left(t\right),\;z^{*}\left(t\right)$ is another solution with initial conditions (x(0),y(0),z(0)) such that by (3), we have

$$x^{*}\left(t\right)=x\left(0\right)=\frac{\beta }{\Gamma \left(\alpha \right)}\int_{0}^{t}U\left(x^{*}\left(\tau \right),y^{*}\left(\tau \right),\;z^{*}\left(\tau \right)\right){\left(t-\tau \right)}^{\alpha -1}\;\tau^{\beta -1}d\tau ,$$

$$y^{*}\left(t\right)=y\left(0\right)=\frac{\beta }{\Gamma \left(\alpha \right)}\int_{0}^{t}V\left(x^{*}\left(\tau \right),y^{*}\left(\tau \right),\;z^{*}\left(\tau \right)\right){\left(t-\tau \right)}^{\alpha -1}\;\tau^{\beta -1}d\tau ,$$

$$z^{*}\left(t\right)=z\left(0\right)=\frac{\beta }{\Gamma \left(\alpha \right)}\int_{0}^{t}W\left(x^{*}\left(\tau \right),y^{*}\left(\tau \right),\;z^{*}\left(\tau \right)\right){\left(t-\tau \right)}^{\alpha -1}\;\tau^{\beta -1}d\tau ,$$

Now, we can estimate

$$\begin{array}{ccc} |x\left(t\right)-x^{*}\left(t\right)| & & \leq \frac{\beta }{\Gamma (\alpha )}\int_{0}^{t}\tau^{\beta -1}\;{\left(t-\tau \right)}^{\alpha -1}\left|U\left(x(\tau ),y(\tau ),\;z(\tau ),\tau \right)-U\left(x^{*}\left(\tau \right),y^{*}\left(\tau \right),\;z^{*}\left(\tau \right),\tau \right)\right|d\tau \\ & & \leq \frac{\beta }{\Gamma (\alpha )}\int_{0}^{t}\tau^{\beta -1}\;{\left(t-\tau \right)}^{\alpha -1}w_{1}∥x\left(t\right)-x^{*}\left(t\right)\|d\tau \leq \frac{\beta t^{\beta +\alpha -1}\Gamma \left(\beta \right)}{\Gamma (\alpha +\beta )}w_{1}∥x\left(t\right)-x^{*}\left(t\right)∥. \end{array}$$

so

$$\begin{array}{c} \left[1-\frac{\beta t^{\beta +\alpha -1}\Gamma \left(\beta \right)}{\Gamma (\alpha +\beta )}w_{1}\right]∥x\left(t\right)-x^{*}\left(t\right)∥\leq 0. \end{array}$$

The latter inequality is true if $∥x\left(t\right)-x^{*}\left(t\right)∥=0$, and accordingly $x\left(t\right)=x^{*}\left(t\right)$. Similarly, one obtains $y\left(t\right)=y^{*}\left(t\right)$ and $z\left(t\right)=z^{*}\left(t\right).$ Consequently, we get $\left(x,\;y,\;z\right)\;=\left(x^{*},\;y^{*},\;z^{*}\right).$ This shows that the fractal-fractional model (1) has a unique solution, and this completes our proof.

### Local stability analysis

For a specific value of the parameters such as a=k=g=10 and d=13ord=4.272 with the initial value (0.1,0.1,0.1), which makes the Burke-Shaw attractor chaotic. The Jacobian matrix J of the Burke-Shaw system for the equilibrium point $E^{*}\;=\;\left(x^{*},\;y^{*},\;z^{*}\right)$ is defined as follows:

$$\begin{array}{c} J=\left[\begin{array}{c} -a\;-a\;0\\ -k\;z^{*}\;-1\;-k\;x^{*}\\ g\;y^{*}\;g\;x^{*}\;0 \end{array}\right]. \end{array}$$

Let *a* = *k* = *g* = 10 and *d* = 13. We first determine the equilibrium points satisfying the following

$$\begin{array}{ccc} 0\; & = & \;-10\;\left(x\left(t\right)\;+\;y\left(t\right)\right),\\ 0\; & = & \;-10\;x\left(t\right)\;z\left(t\right)\;-\;y\left(t\right),\\ 0\; & = & \;10\;x\left(t\right)\;y\left(t\right)\;+\;13. \end{array}$$

The Burke-Shaw system (1) with above parameters has two equilibrium points: *E*_1_ = (1*.*1402*, −*1*.*1402*,* 0*.*1) and *E*_2_ = (*−*1*.*1402*,* 1*.*1402*,* 0*.*1). Obviously, due to parameter d in the model there is no equilibrium point at the origin. For the equilibrium points *E*_1_, *E*_2_, we obtain the same eigenvalues.

*λ*_1_*≈ −*14*.*4527 and *λ*_2_*_,_*_3_*≈* 1*.*7263 *±* 13*.*3013*i*. Both equilibria are unstable.

### Lyapunov exponents

Now use the Danca algorithm [1,2] and use the Adams-Bashforth-Moulton numerical scheme to compute the Lyapunov exponent (LE) of (1.2). From Table 2, we can see that system (1) is dissipative since the sum of the Lyapunov exponents (LE) in each row of the table is negative. Note that the Lyapunov exponent *α*(t) depends on x(t)=0.1,y(t)=0.1, and z(t)=0.1.

### Kaplan-Yorke dimension

Here are the Kaplan-Yorke dimensions for some of the fractional derivatives presented in Table 1:

$$\text{dim}\left(LE\right)=\;2\;+\frac{LE_{1}+LE_{2}}{\left|LE_{3}\right|}.$$

$$\begin{array}{ccc} \text{For}\;\alpha =0.70, & & \;\text{dim}\left(LE\right)=\;2+\frac{6.7601\;-\;0.0781}{\left|-\;53.6901\right|}=\;0.1273.\\ \text{For}\;\alpha =0.90, & & \;\text{dim}\left(LE\right)=\;2+\frac{3.5788\;-\;0.0056}{\left|-\;21.6809\right|}=0.1653.\\ \text{For}\;\alpha =0.98, & & \;\text{dim}\left(LE\right)=\;2+\frac{2.4723\;-\;0.0060}{\left|-\;14.6040\right|}=\;0.1688.\\ \text{For}\;\alpha =1, & & \;\text{dim}\left(LE\right)=\;2+\frac{2.2695\;-\;0.0069}{\left|-\;13.2372\right|}=\;0.1709. \end{array}$$

**Table 1:** Equilibrium points and corresponding eigenvalues.

Equilibrium points	Eigenvalues	Nature	Index
*E*_1_ = (1*.*1402*, −*1*.*1402*,* 0*.*1)	*−*14*.*4527, 1*.*7263 + 13*.*3013*i*,	saddle-focus point	1
*E*_2_ = (*−*1*.*1402*,* 1*.*1402*,* 0*.*1)	*−*14*.*4527, 1*.*7263 *−* 13*.*3013*i*,	saddle-focus point	1

**Table 2:** Lyapunov exponents versus *α* of a fractional Newton-Leipnik system (1).

*α*	LE1	LE2	LE3
0.7	6.7601	−0.0781	−53.6901
0.9	3.5788	−0.0056	−21.6809
0.98	2.4723	−0.0060	−14.6040
1	2.2695	−0.0069	−13.2372

**Fig. 1:**
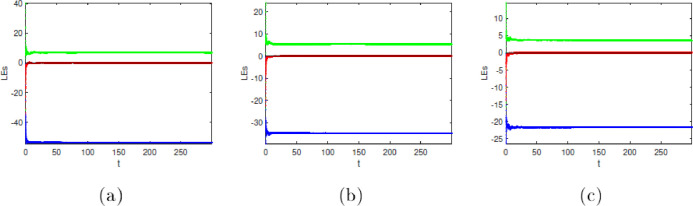
Dynamics of the system (1) in(x,y),(x,z),(y,z),and (x,y,z) planes with *α*(t) = 1, respectively in (A), (B), (C), (D).

The fact that all the Kaplan-Yorke dimensions calculated earlier are fractional is another indication that the system is moving in a chaotic direction. Fig. 1 simulation results demonstrate the Lyapunov exponential spectrum technique for chaotic fractional-order systems high accuracy and convergence.

### Dissipativity

The divergent flow of (1) is dissipative if and only if ∇V<0*,*

∇V=−a−0.4+b.

If b−a<0.4 then the system is dissipative.

### Symmetry

Since the system (1) is invariant under the coordinate transformation (x,y,z)→(−x,−y,z), the system (1) is symmetric about the z axis.

### Numerical scheme of fractal-fractional with power-law kernel

In this section, we now consider our model with Caputo fractal-fractional operator as in Atangana- Seda numerical scheme [40]:

$$\begin{array}{ccc} {}^{\text{FF}}D_{0,t}^{\alpha ,\beta }x\left(t\right) & = & -a\left(x\left(t\right)+\;y\left(t\right)\right),\\ {}^{\text{FF}}D_{0,t}^{\alpha ,\;\beta }y\left(t\right) & = & -k\;x\left(t\right)\;z\left(t\right)-y\left(t\right),\\ {}^{\text{FF}}D_{0,t}^{\alpha ,\beta }z\left(t\right) & = & \;g\;x(t)\;y(t)\;+\;d. \end{array}$$

We can write as follows for simplicity

$$\begin{array}{ccc} {}^{\text{FF}}D_{0,t}^{\alpha ,\beta }x\left(t\right) & = & \varphi \;\left(x,\;y,\;z,t\right),\\ {}^{\text{FF}}D_{0,t}^{\alpha ,\beta }y\left(t\right) & = & \;\psi \left(x,\;y,\;z,t\right),\\ {}^{\text{FF}}D_{0,t}^{\alpha ,\beta }z\left(t\right) & = & \;\mu (x,\;y,\;z). \end{array}$$

and we reorder the above equation as follows:

$$\begin{array}{ccc} {}^{C}D_{0,t}^{\alpha }x\left(t\right) & = & \beta \;t^{\beta -1}\varphi \;\left(x,\;y,\;z,t\right),\\ {}^{C}D_{0,t}^{\alpha }y\left(t\right) & = & \beta \;t^{\beta -1}\;\psi \left(x,\;y,\;z,t\right),\\ {}^{C}D_{0,t}^{\alpha }z\left(t\right) & = & \beta \;t^{\beta -1}\;\mu \left(x,\;y,\;z\right). \end{array}$$

Taking as

$$\begin{array}{ccc} U\left(x,\;y,\;z,t\right) & = & \beta \;t^{\beta -1}\varphi \;\left(x,\;y,\;z,t\right),\\ V\left(x,\;y,\;z,t\right) & = & \beta \;t^{\beta -1}\;\psi \left(x,\;y,\;z,t\right),\\ W(x,\;y,\;z,t) & = & \beta \;t^{\beta -1}\;\mu \left(x,\;y,\;z\right). \end{array}$$

and calculating the integral of above equation, we can get the following:

$$\begin{array}{ccc} x\left(t\right) & = & x\left(0\right)+\frac{1}{\Gamma \left(\alpha \right)}\int_{0}^{t}U\left(x\left(\tau \right),y\left(\tau \right),\;z\left(\tau \right),\tau \right){\left(t-\tau \right)}^{\alpha -1}\;d\tau ,\\ y\left(t\right) & = & y\left(0\right)+\frac{1}{\Gamma \left(\alpha \right)}\int_{0}^{t}V\left(x\left(\tau \right),y\left(\tau \right),\;z\left(\tau \right),\tau \right){\left(t-\tau \right)}^{\alpha -1}\;d\tau ,\\ z\left(t\right) & = & z\left(0\right)+\frac{1}{\Gamma \left(\alpha \right)}\int_{0}^{t}W\left(x\left(\tau \right),y\left(\tau \right),\;z\left(\tau \right),\tau \right){\left(t-\tau \right)}^{\alpha -1}\;d\tau , \end{array}$$

We have at point $t=t_{n+1}$

$$\begin{array}{ccc} x\left(t_{n+1}\right) & = & x\left(0\right)+\frac{1}{\Gamma \left(\alpha \right)}\int_{0}^{t_{n+1}}U\left(x\left(\tau \right),y\left(\tau \right),\;z\left(\tau \right),\tau \right){\left(t_{n+1}-\tau \right)}^{\alpha -1}\;d\tau ,\\ y\left(t_{n+1}\right) & = & y\left(0\right)+\frac{1}{\Gamma \left(\alpha \right)}\int_{0}^{t_{n+1}}V\left(x\left(\tau \right),y\left(\tau \right),\;z\left(\tau \right),\tau \right){\left(t_{n+1}-\tau \right)}^{\alpha -1}\;d\tau ,\\ z\left(t_{n+1}\right) & = & z\left(0\right)+\frac{1}{\Gamma \left(\alpha \right)}\int_{0}^{t_{n+1}}W\left(x\left(\tau \right),y\left(\tau \right),\;z\left(\tau \right),\tau \right){\left(t_{n+1}-\tau \right)}^{\alpha -1}\;d\tau , \end{array}$$

and we write

$$\begin{array}{ccc} x\left(t_{n+1}\right) & = & x\left(t\right)+\frac{1}{\Gamma \left(\alpha \right)}\sum_{r=2}^{n}\int_{t_{r}}^{t_{r+1}}U\left(x\left(\tau \right),y\left(\tau \right),\;z\left(\tau \right),\tau \right){\left(t_{n+1}-\tau \right)}^{\alpha -1}\;d\tau ,\\ y\left(t_{n+1}\right) & = & y\left(t\right)+\frac{1}{\Gamma \left(\alpha \right)}\sum_{r=2}^{n}\int_{t_{r}}^{t_{r+1}}V\left(x\left(\tau \right),y\left(\tau \right),\;z\left(\tau \right),\tau \right){\left(t_{n+1}-\tau \right)}^{\alpha -1}\;d\tau ,\\ z\left(t_{n+1}\right) & = & z\left(t\right)+\frac{1}{\Gamma \left(\alpha \right)}\sum_{r=2}^{n}\int_{t_{r}}^{t_{r+1}}W\left(x\left(\tau \right),y\left(\tau \right),\;z\left(\tau \right),\tau \right){\left(t_{n+1}-\tau \right)}^{\alpha -1}\;d\tau , \end{array}$$

If we use two step Newton polynomial, we write the following:

$$\begin{array}{ccc} x\left(t_{n+1}\right) & = & x\left(t\right)+\frac{1}{\Gamma \left(\alpha \right)}\sum_{r=2}^{n}\int_{t_{r}}^{t_{r+1}}\{U\left(x^{r-2},y^{r-2},\;z^{r-2},t_{r-2}\right)+\frac{U\left(x^{r-1},y^{r-1},\;z^{r-1},t_{r-1}\right)-U\left(x^{r-2},y^{r-2},\;z^{r-2},t_{r-2}\right)}{\Delta t}\left(\tau -t_{r-2}\right)\\ & & +\frac{U\left(x^{r},y^{r},\;z^{r},t_{r}\right)-2U\left(x^{r-1},y^{r-1},\;z^{r-1},t_{r-1}\right)+U\left(x^{r-2},y^{r-2},\;z^{r-2},t_{r-2}\right)}{2{\left(\Delta t\right)}^{2}}\times \left(\tau -t_{r-2}\right)\left(\tau -t_{r-1}\right)\}{\left(t_{n+1}-\tau \right)}^{\alpha -1}\;d\tau , \end{array}$$

$$\begin{array}{ccc} y\left(t_{n+1}\right) & = & y\left(t\right)+\frac{1}{\Gamma \left(\alpha \right)}\sum_{r=2}^{n}\int_{t_{r}}^{t_{r+1}}\{V\left(x^{r-2},y^{r-2},\;z^{r-2},t_{r-2}\right)+\frac{V\left(x^{r-1},y^{r-1},\;z^{r-1},t_{r-1}\right)-V\left(x^{r-2},y^{r-2},\;z^{r-2},t_{r-2}\right)}{\Delta t}\left(\tau -t_{r-2}\right)\\ & & +\frac{V\left(x^{r},y^{r},\;z^{r},t_{r}\right)-2V\left(x^{r-1},y^{r-1},\;z^{r-1},t_{r-1}\right)+V\left(x^{r-2},y^{r-2},\;z^{r-2},t_{r-2}\right)}{2{\left(\Delta t\right)}^{2}}\times \left(\tau -t_{r-2}\right)\left(\tau -t_{r-1}\right)\}{\left(t_{n+1}-\tau \right)}^{\alpha -1}\;d\tau \end{array}$$

$$\begin{array}{ccc} z\left(t_{n+1}\right) & = & z\left(t\right)+\frac{1}{\Gamma \left(\alpha \right)}\sum_{r=2}^{n}\int_{t_{r}}^{t_{r+1}}\{W\left(x^{r-2},y^{r-2},\;z^{r-2},t_{r-2}\right)+\frac{W\left(x^{r-1},y^{r-1},\;z^{r-1},t_{r-1}\right)-W\left(x^{r-2},y^{r-2},\;z^{r-2},t_{r-2}\right)}{\Delta t}\left(\tau -t_{r-2}\right)\\ & & +\frac{W\left(x^{r},y^{r},\;z^{r},t_{r}\right)-2W\left(x^{r-1},y^{r-1},\;z^{r-1},t_{r-1}\right)+W\left(x^{r-2},y^{r-2},\;z^{r-2},t_{r-2}\right)}{2{\left(\Delta t\right)}^{2}}\times \left(\tau -t_{r-2}\right)\left(\tau -t_{r-1}\right)\}{\left(t_{n+1}-\tau \right)}^{\alpha -1}\;d\tau , \end{array}$$

and if we organize the above equations, we can have the following.

$$\begin{array}{ccc} x^{n+1} & = & x^{0}+\frac{1}{\Gamma \left(\alpha \right)}\sum_{r=2}^{n}U\left(x^{r-2},y^{r-2},\;z^{r-2},t_{r-2}\right)\int_{t_{r}}^{t_{r+1}}{\left(t_{n+1}-\tau \right)}^{\alpha -1}\;d\tau \\ & & +\frac{1}{\Gamma \left(\alpha \right)}\sum_{r=2}^{n}\frac{U\left(x^{r-1},y^{r-1},\;z^{r-1},t_{r-1}\right)-U\left(x^{r-2},y^{r-2},\;z^{r-2},t_{r-2}\right)}{\Delta t}\int_{t_{r}}^{t_{r+1}}\left(\tau -t_{r-2}\right){\left(t_{n+1}-\tau \right)}^{\alpha -1}\;d\tau \\ & & +\frac{1}{\Gamma \left(\alpha \right)}\sum_{r=2}^{n}\frac{U\left(x^{r},y^{r},\;z^{r},t_{r}\right)-2U\left(x^{r-1},y^{r-1},\;z^{r-1},t_{r-1}\right)+U\left(x^{r-2},y^{r-2},\;z^{r-2},t_{r-2}\right)}{2{\left(\Delta t\right)}^{2}}\int_{t_{r}}^{t_{r+1}}\left(\tau -t_{r-1}\right)\left(\tau -t_{r-2}\right){\left(t_{n+1}\;-\;\tau \right)}^{\alpha -1}\;d\tau , \end{array}$$

$$\begin{array}{ccc} y^{n+1} & = & y^{0}+\frac{1}{\Gamma \left(\alpha \right)}\sum_{r=2}^{n}V\left(x^{r-2},y^{r-2},\;z^{r-2},t_{r-2}\right)\int_{t_{r}}^{t_{r+1}}{\left(t_{n+1}-\tau \right)}^{\alpha -1}\;d\tau \\ & & +\frac{1}{\Gamma \left(\alpha \right)}\sum_{r=2}^{n}\frac{V\left(x^{r-1},y^{r-1},\;z^{r-1},t_{r-1}\right)-V\left(x^{r-2},y^{r-2},\;z^{r-2},t_{r-2}\right)}{\Delta t}\int_{t_{r}}^{t_{r+1}}\left(\tau -t_{r-2}\right){\left(t_{n+1}-\tau \right)}^{\alpha -1}\;d\tau \\ & & +\frac{1}{\Gamma \left(\alpha \right)}\sum_{r=2}^{n}\frac{V\left(x^{r},y^{r},\;z^{r},t_{r}\right)-2V\left(x^{r-1},y^{r-1},\;z^{r-1},t_{r-1}\right)+V\left(x^{r-2},y^{r-2},\;z^{r-2},t_{r-2}\right)}{2{\left(\Delta t\right)}^{2}}\int_{t_{r}}^{t_{r+1}}\left(\tau -t_{r-1}\right)\left(\tau -t_{r-2}\right){\left(t_{n+1}\;-\;\tau \right)}^{\alpha -1}\;d\tau , \end{array}$$

$$\begin{array}{ccc} z^{n+1} & = & z^{0}+\frac{1}{\Gamma (\alpha )}\sum_{r=2}^{n}U\left(x^{r-2},y^{r-2},\;z^{r-2},t_{r-2}\right)\int_{t_{r}}^{t_{r+1}}{\left(t_{n+1}-\tau \right)}^{\alpha -1}\;d\tau +\frac{1}{\Gamma (\alpha )}\sum_{r=2}^{n}\frac{W\left(x^{r-1},y^{r-1},\;z^{r-1},t_{r-1}\right)-W\left(x^{r-2},y^{r-2},\;z^{r-2},t_{r-2}\right)}{\Delta t}\\ & & \int_{t_{r}}^{t_{r+1}}\left(\tau -t_{r-2}\right){\left(t_{n+1}-\tau \right)}^{\alpha -1}\;d\tau +\frac{1}{\Gamma (\alpha )}\sum_{r=2}^{n}\frac{W\left(x^{r},y^{r},\;z^{r},t_{r}\right)-2W\left(x^{r-1},y^{r-1},\;z^{r-1},t_{r-1}\right)+W\left(x^{r-2},y^{r-2},\;z^{r-2},t_{r-2}\right)}{2{\left(\Delta t\right)}^{2}}\\ & & \int_{t_{r}}^{t_{r+1}}\left(\tau -t_{r-1}\right)\left(\tau -t_{r-2}\right){\left(t_{n+1}-\tau \right)}^{\alpha -1}\;d\tau . \end{array}$$

We have the following calculation.

$$\int_{t_{r}}^{t_{r+1}}{\left(t_{n+1}-\tau \right)}^{\alpha -1}\;d\tau =\frac{{\left(\Delta t\right)}^{\alpha }}{\alpha }\left[{\left(n-r+1\right)}^{\alpha }-{\left(n-r\right)}^{\alpha }\right],$$

$$\int_{t_{r}}^{t_{r+1}}\left(\tau -t_{r-1}\right){\left(t_{n+1}-\tau \right)}^{\alpha -1}\;d\tau =\frac{{\left(\Delta t\right)}^{\alpha +1}}{\alpha \left(\alpha +1\right)}\left[{\left(n-r+1\right)}^{\alpha }\left(n-r+3+2\alpha \right)-{\left(n-r\right)}^{\alpha }\left(n-r+3+3\alpha \right)\right],$$

$$\begin{array}{ccc} & & \int_{t_{r}}^{t_{r+1}}\left(\tau -t_{r-1}\right)\left(\tau -t_{r-2}\right){\left(t_{n+1}-\tau \right)}^{\alpha -1}\;d\tau \\ & & =\frac{{\left(\Delta t\right)}^{\alpha +1}}{\alpha (\alpha +1)(\alpha +2)}\left[{\left(n-r+1\right)}^{\alpha }\left(2{\left(n-r\right)}^{2}+\left(3\alpha +10\right)\left(n-r\right)+2\alpha^{2}+12+9\alpha \right)-{\left(n-r\right)}^{\alpha }\left(2{\left(n-r\right)}^{2}+\left(5\alpha +10\right)\left(n-r\right)+6\alpha^{2}+12+18\alpha \right)\right]. \end{array}$$

Thus, numerical solution of Burk-Shaw is given the following scheme.

$$\begin{array}{ccc} x^{n+1} & = & x^{0}+\frac{{\left(\Delta t\right)}^{\alpha }}{\Gamma \left(\alpha +1\right)}\sum_{r=2}^{n}\beta \;t_{r-2}^{\beta -1}\varphi \left(x^{r-2},y^{r-2},\;z^{r-2},t_{r-2}\right)\left[{\left(n-r+1\right)}^{\alpha }-{\left(n-r\right)}^{\alpha }\right]\\ & & +\frac{{\left(\Delta t\right)}^{\alpha }}{\Gamma \left(\alpha +2\right)}\sum_{r=2}^{n}\left[\beta \;t_{r-1}^{\beta -1}\varphi \left(x^{r-1},y^{r-1},\;z^{r-1},t_{r-1}\right)-\beta \;t_{r-2}^{\beta -1}\varphi \left(x^{r-2},y^{r-2},\;z^{r-2},t_{r-2}\right)\right]\left[{\left(n-r+1\right)}^{\alpha }\left(n-r+3+2\alpha \right)-{\left(n-r\right)}^{\alpha }\left(n-r+3+3\alpha \right)\right]\\ & & +\frac{{\left(\Delta t\right)}^{\alpha +1}}{\Gamma \left(\alpha +3\right)}\sum_{r=2}^{n}\left[\beta \;t_{r}^{\beta -1}\varphi \left(x^{r},y^{r},\;z^{r},t_{r}\right)-2\beta \;t_{r-1}^{\beta -1}\varphi \left(x^{r-1},y^{r-1},\;z^{r-1},t_{r-1}\right)+\beta \;t_{r-2}^{\beta -1}\varphi \left(x^{r-2},y^{r-2},\;z^{r-2},t_{r-2}\right)\right]\\ & & \left[{\left(n-r+1\right)}^{\alpha }\left(2{\left(n-r\right)}^{2}+\left(3\alpha +10\right)\left(n-r\right)+2\alpha^{2}+12+9\alpha \right)-{\left(n-r\right)}^{\alpha }\left(2{\left(n-r\right)}^{2}+\left(5\alpha +10\right)\left(n-r\right)+6\alpha^{2}+12+18\alpha \right)\right], \end{array}$$

$$\begin{array}{ccc} y^{n+1} & = & y^{0}+\frac{{\left(\Delta t\right)}^{\alpha }}{\Gamma \left(\alpha +1\right)}\sum_{r=2}^{n}\beta \;t_{r-2}^{\beta -1}\psi \left(x^{r-2},y^{r-2},\;z^{r-2},t_{r-2}\right)\left[{\left(n-r+1\right)}^{\alpha }-{\left(n-r\right)}^{\alpha }\right]\\ & & +\frac{{\left(\Delta t\right)}^{\alpha }}{\Gamma \left(\alpha +2\right)}\sum_{r=2}^{n}\left[\beta \;t_{r-1}^{\beta -1}\psi \left(x^{r-1},y^{r-1},\;z^{r-1},t_{r-1}\right)-\beta \;t_{r-2}^{\beta -1}\psi \left(x^{r-2},y^{r-2},\;z^{r-2},t_{r-2}\right)\right]\left[{\left(n-r+1\right)}^{\alpha }\left(n-r+3+2\alpha \right)-{\left(n-r\right)}^{\alpha }\left(n-r+3+3\alpha \right)\right]\\ & & +\frac{{\left(\Delta t\right)}^{\alpha +1}}{\Gamma \left(\alpha +3\right)}\sum_{r=2}^{n}\left[\beta \;t_{r}^{\beta -1}\psi \left(x^{r},y^{r},\;z^{r},t_{r}\right)-2\beta \;t_{r-1}^{\beta -1}\psi \left(x^{r-1},y^{r-1},\;z^{r-1},t_{r-1}\right)+\beta \;t_{r-2}^{\beta -1}\psi \left(x^{r-2},y^{r-2},\;z^{r-2},t_{r-2}\right)\right]\\ & & \left[{\left(n-r+1\right)}^{\alpha }\left(2{\left(n-r\right)}^{2}+\left(3\alpha +10\right)\left(n-r\right)+2\alpha^{2}+12+9\alpha \right)-{\left(n-r\right)}^{\alpha }\left(2{\left(n-r\right)}^{2}+\left(5\alpha +10\right)\left(n-r\right)+6\alpha^{2}+12+18\alpha \right)\right], \end{array}$$

$$\begin{array}{ccc} z^{n+1} & = & z^{0}+\frac{{\left(\Delta t\right)}^{\alpha }}{\Gamma \left(\alpha +1\right)}\sum_{r=2}^{n}\beta \;t_{r-2}^{\beta -1}\mu \left(x^{r-2},y^{r-2},\;z^{r-2},t_{r-2}\right)\left[{\left(n-r+1\right)}^{\alpha }-{\left(n-r\right)}^{\alpha }\right]\\ & & +\frac{{\left(\Delta t\right)}^{\alpha }}{\Gamma \left(\alpha +2\right)}\sum_{r=2}^{n}\left[\beta \;t_{r-1}^{\beta -1}\mu \left(x^{r-1},y^{r-1},\;z^{r-1},t_{r-1}\right)-\beta \;t_{r-2}^{\beta -1}\mu \left(x^{r-2},y^{r-2},\;z^{r-2},t_{r-2}\right)\right]\left[{\left(n-r+1\right)}^{\alpha }\left(n-r+3+2\alpha \right)-{\left(n-r\right)}^{\alpha }\left(n-r+3+3\alpha \right)\right]\\ & & +\frac{{\left(\Delta t\right)}^{\alpha +1}}{\Gamma \left(\alpha +3\right)}\sum_{r=2}^{n}\left[\beta \;t_{r}^{\beta -1}\mu \left(x^{r},y^{r},\;z^{r},t_{r}\right)-2\beta \;t_{r-1}^{\beta -1}\mu \left(x^{r-1},y^{r-1},\;z^{r-1},t_{r-1}\right)+\beta \;t_{r-2}^{\beta -1}\mu \left(x^{r-2},y^{r-2},\;z^{r-2},t_{r-2}\right)\right]\\ & & \left[{\left(n-r+1\right)}^{\alpha }\left(2{\left(n-r\right)}^{2}+\left(3\alpha +10\right)\left(n-r\right)+2\alpha^{2}+12+9\alpha \right)-{\left(n-r\right)}^{\alpha }\left(2{\left(n-r\right)}^{2}+\left(5\alpha +10\right)\left(n-r\right)+6\alpha^{2}+12+18\alpha \right)\right], \end{array}$$

### Numerical scheme of variable order fractal-fractional with power-law kernel

Let u be a differential function. Let α be a constant fractional order such that 0<α≤1. Let β(t)>0 be continuous function, then a fractional derivative of u with order α and fractal variable dimension β(t) is given by

$${}^{\text{FF}}D_{0,t}^{\alpha ,\beta \left(t\right)}u\left(t\right)=\frac{1}{\Gamma \left(1-\alpha \right)}\frac{d}{dt^{\beta \left(t\right)}}\int_{0}^{t}{\left(t-\tau \right)}^{\alpha -1}u\left(\tau \right)\;d\tau$$

Where

$$\frac{d\gamma \left(\tau \right)}{dt^{\beta \left(t\right)}}=\lim_{t\to \tau }\frac{\gamma \left(t\right)-\gamma \left(\tau \right)}{t^{\beta \left(t\right)}-\tau^{\beta \left(t\right)}}.$$

The new fractional integral with power-law kernel is given by

$${}^{\text{FF}}I_{0,t}^{\alpha ,\beta \left(t\right)}u\left(t\right)=\frac{1}{\Gamma \left(\alpha \right)}\int_{0}^{t}{\left(t-\tau \right)}^{\alpha -1}u\left(\tau \right)\left[\beta^{\prime }\left(\tau \right)\ln\left(\tau \right)+\frac{\beta \left(\tau \right)}{\tau }\right]\;\tau^{\beta \left(\tau \right)}d\tau .$$

In this section, we give the derivation of a numerical solution of the following problem:

$${}^{\text{FF}}D_{0,t}^{\alpha ,\beta \left(t\right)}u\left(t\right)=h\left(t,u\left(t\right)\right),$$

$u\left(0\right)=u_{0}.$

Applying the new fractional integral with power kernel, we can rewrite the above equation as

$u\left(t\right)=\frac{1}{\Gamma (\alpha )}\int_{0}^{t}{\left(t-\tau \right)}^{\alpha -1}h\left(t,u(t)\right)\left[\beta^{\prime }\left(\tau \right)\ln\left(\tau \right)+\frac{\beta (\tau )}{\tau }\right]\;\tau^{\beta (\tau )}d\tau .$

At the point $t_{k+1}\;=\;\left(k\;+\;1\right)\Delta t$, we can have the following:

$$u\left(t_{k+1}\right)-u\left(t\right)=\frac{1}{\Gamma \left(\alpha \right)}\int_{0}^{t_{k+1}}{\left(t_{k+1}-\tau \right)}^{\alpha -1}g\left(\tau ,u\left(\tau \right)\right)d\tau ,$$

where

$$g\left(\tau ,u\left(\tau \right)\right)=h\left(\tau ,u\left(\tau \right)\right)\left[\beta^{\prime }\left(\tau \right)\ln\left(\tau \right)+\frac{\beta \left(\tau \right)}{\tau }\right]\;\tau^{\beta \left(\tau \right)}.$$

Then, we have

$$u\left(t_{k+1}\right)=u_{0}+\frac{1}{\Gamma \left(\alpha \right)}\int_{0}^{t_{k+1}}{\left(t_{k+1}-\tau \right)}^{\alpha -1}g\left(\tau ,u\left(\tau \right)\right)d\tau ,$$

and we write

$$u\left(t_{k+1}\right)=u_{0}+\frac{1}{\Gamma \left(\alpha \right)}\sum_{m=0}^{k}\int_{t_{m}}^{t_{m+1}}{\left(t_{k+1}-\tau \right)}^{\alpha -1}g\left(\tau ,u\left(\tau \right)\right)d\tau .$$

Using the Lagrange polynomial, the above equation can be revised,

(4) $$u\left(t_{k+1}\right)=u_{0}+\frac{1}{\Gamma \left(\alpha \right)}\sum_{m=0}^{k}\int_{t_{m}}^{t_{m+1}}\left\{\frac{g\left(t_{m},x^{m}\right)}{\Delta t}\left(\tau -t_{m+1}\right)-\frac{g\left(t_{m-1},x^{m-1}\right)}{\Delta t}\left(\tau -t_{m}\right)\right\}{\left(t_{k+1}-\tau \right)}^{\alpha -1}d\tau .$$

Thus, we have

$$u_{k+1}=u_{0}+\frac{1}{\Gamma \left(\alpha \right)}\sum_{m=0}^{k}\int_{t_{m}}^{t_{m+1}}\frac{g\left(t_{m},x^{m}\right)}{\Delta t}\left(\tau -t_{m+1}\right){\left(t_{k+1}-\tau \right)}^{\alpha -1}d\tau -\int_{t_{m}}^{t_{m+1}}\frac{g\left(t_{m-1},x^{m-1}\right)}{\Delta t}\left(\tau -t_{m}\right){\left(t_{k+1}-\tau \right)}^{\alpha -1}d\tau .$$

And

$$u_{k+1}=u_{0}+\frac{1}{\Gamma \left(\alpha \right)}\sum_{m=0}^{k}\frac{g\left(t_{m},x^{m}\right)}{\Delta t}\int_{t_{m}}^{t_{m+1}}\left(\tau -t_{m+1}\right){\left(t_{k+1}-\tau \right)}^{\alpha -1}d\tau -\frac{1}{\Gamma \left(\alpha \right)}\sum_{m=0}^{k}\frac{g\left(t_{m-1},x^{m-1}\right)}{\Delta t}\int_{t_{m}}^{t_{m+1}}\left(\tau -t_{m}\right){\left(t_{k+1}-\tau \right)}^{\alpha -1}d\tau .$$

We put the calculations for the above integrals into Eq. (4), and we obtain the following approximation.

$$\int_{t_{r}}^{t_{r+1}}{\left(t_{n+1}-\tau \right)}^{\alpha -1}\;d\tau =\frac{{\left(\Delta t\right)}^{\alpha }}{\alpha }\left[{\left(n-r+1\right)}^{\alpha }-{\left(n-r\right)}^{\alpha }\right],$$

$$\int_{t_{r}}^{t_{r+1}}\left(\tau -t_{r-1}\right){\left(t_{n+1}-\tau \right)}^{\alpha -1}\;d\tau =\frac{{\left(\Delta t\right)}^{\alpha +1}}{\alpha \left(\alpha +1\right)}\left[{\left(n-r+1\right)}^{\alpha }\left(n-r+3+2\alpha \right)-{\left(n-r\right)}^{\alpha }\left(n-r+3+3\alpha \right)\right],$$

$$\begin{array}{ccc} & & \int_{t_{r}}^{t_{r+1}}\left(\tau -t_{r-1}\right)\left(\tau -t_{r-2}\right){\left(t_{n+1}-\tau \right)}^{\alpha -1}\;d\tau \\ & & =\frac{{\left(\Delta t\right)}^{\alpha +1}}{\alpha (\alpha +1)(\alpha +2)}\left[{\left(n-r+1\right)}^{\alpha }\left(2{\left(n-r\right)}^{2}+\left(3\alpha +10\right)\left(n-r\right)+2\alpha^{2}+12+9\alpha \right)-{\left(n-r\right)}^{\alpha }\left(2{\left(n-r\right)}^{2}+\left(5\alpha +10\right)\left(n-r\right)+6\alpha^{2}+12+18\alpha \right)\right]. \end{array}$$

$$\begin{array}{ccc} u_{k+1} & = & u_{0}+\frac{{\left(\Delta t\right)}^{\alpha }}{\Gamma \left(\alpha +2\right)}\sum_{m=0}^{k}g\left(t_{m},x^{m}\right)\left[{\left(k-m+1\right)}^{\alpha }\left(k-m+2+\alpha \right)-{\left(k-m\right)}^{\alpha }\left(k-m+2+2\alpha \right)\right]\\ & & -\frac{\alpha {\left(\Delta t\right)}^{\alpha }}{\Gamma \left(\alpha +2\right)}\sum_{m=0}^{k}g\left(t_{m-1},x^{m-1}\right)\left[{\left(k-m+1\right)}^{\alpha +1}-{\left(k-m\right)}^{\alpha }\left(k-m+1+\alpha \right)\right]. \end{array}$$

Thus, if we replace the function g(t,u(t)) by its value, we have the following:

$$\begin{array}{ccc} u_{k+1} & = & u_{0}+\frac{\beta {\left(\Delta t\right)}^{\alpha }}{\Gamma \left(\alpha +2\right)}\sum_{m=0}^{k}\;t_{m}^{\beta \left(t_{m}\right)}\left[\frac{\beta \left(t_{m+1}\right)-\beta \left(t_{m}\right)}{\Delta t}\ln t_{m}+\frac{\beta \left(t_{m}\right)}{t_{m}}\right]h\left(t_{m},x^{m}\right)\left[{\left(k-m+1\right)}^{\alpha }\left(k-m+2+\alpha \right)-{\left(k-m\right)}^{\alpha }\left(k-m+2+2\alpha \right)\right]\\ & & -\frac{\beta {\left(\Delta t\right)}^{\alpha }}{\Gamma \left(\alpha +2\right)}\sum_{m=0}^{k}t_{m-1}^{\beta \left(t_{m-1}\right)}\left[\frac{\beta \left(t_{m}\right)-\beta \left(t_{m-1}\right)}{\Delta t}\ln t_{m-1}+\frac{\beta \left(t_{m-1}\right)}{t_{m-1}}\right]h\left(t_{m-1},x^{m-1}\right)\left[{\left(k-m+1\right)}^{\alpha +1}-{\left(k-m\right)}^{\alpha }\left(k-m+1+\alpha \right)\right]. \end{array}$$

### Numerical simulation

**Fig. 2:**
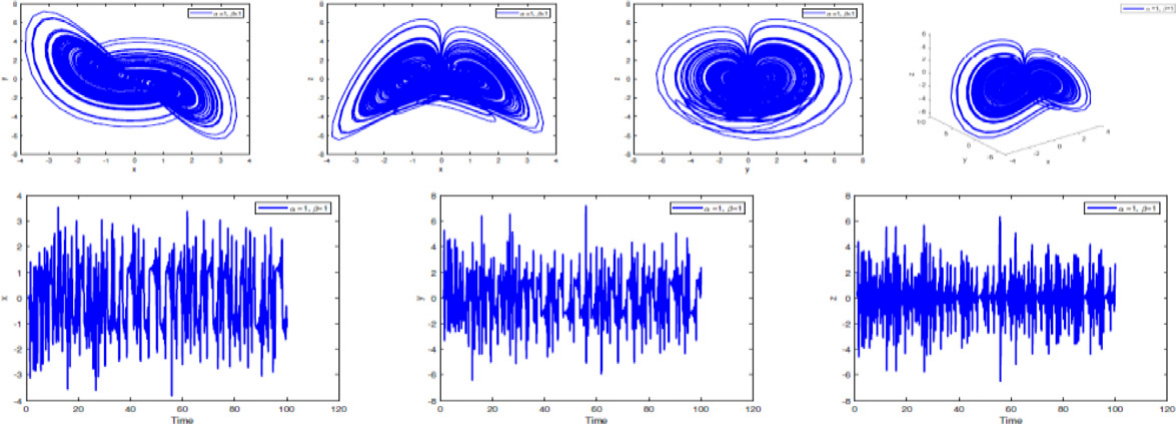
Numerical simulation for fractal fractional Burk-Shaw system using Atangana-Baleanu-Caputo at α=1,β=1.

**Fig. 3:**
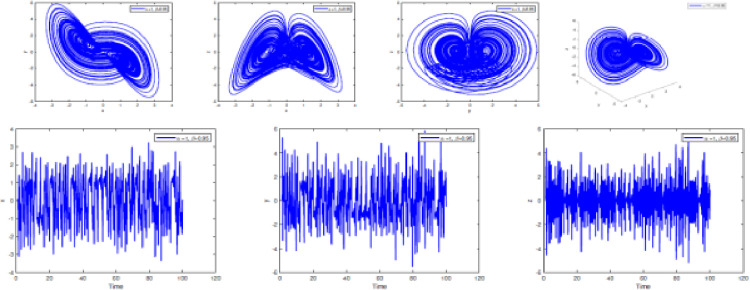
Numerical simulation for fractal fractional Burk-Shaw system using Atangana-Baleanu-Caputo at α=1,β=0.95.

**Fig. 4:**
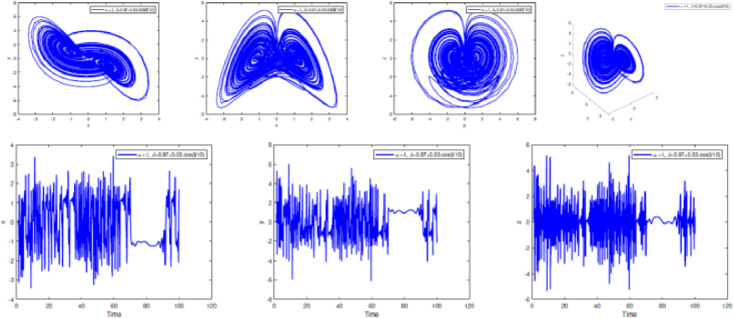
Numerical simulation for fractal fractional Burk-Shaw system using Atangana-Baleanu-Caputo at α=1,β=0.97+0.03.cos(t/10).

**Fig. 5:**
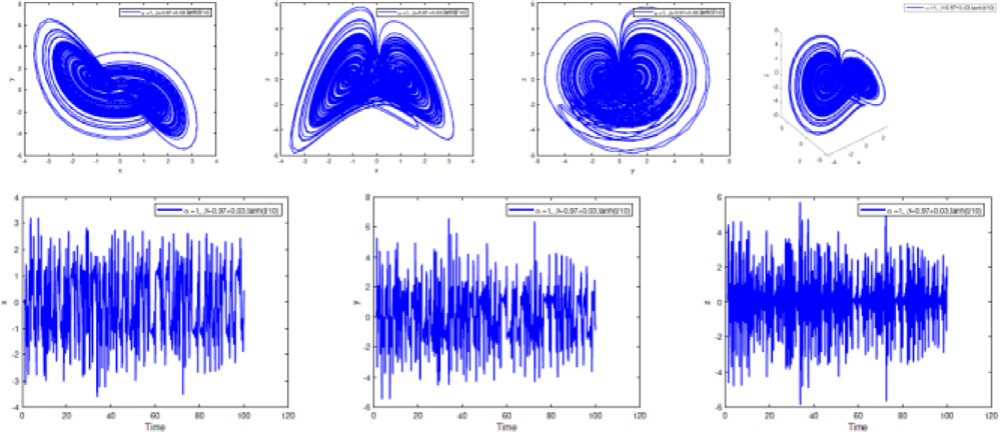
Numerical simulation for fractal fractional Burk-Shaw system using Atangana-Baleanu-Caputo at α=1,β=0.97+0.03.tanh(t/10).

**Fig. 6:**
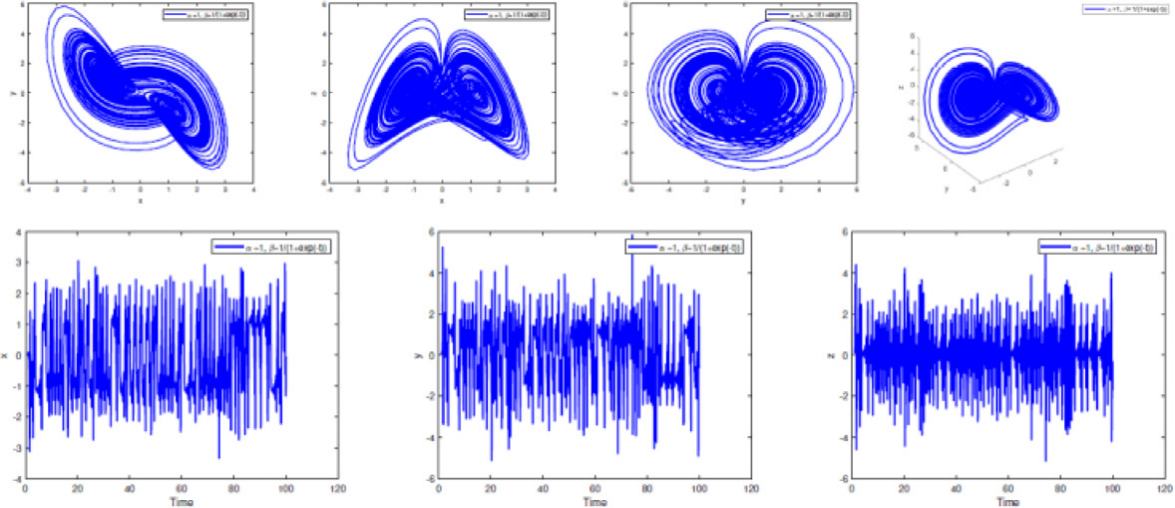
Numerical simulation for fractal fractional Burk-Shaw system using Atangana-Baleanu-Caputo at α=1,β=1/(1+exp(−t)).

**Fig. 7:**
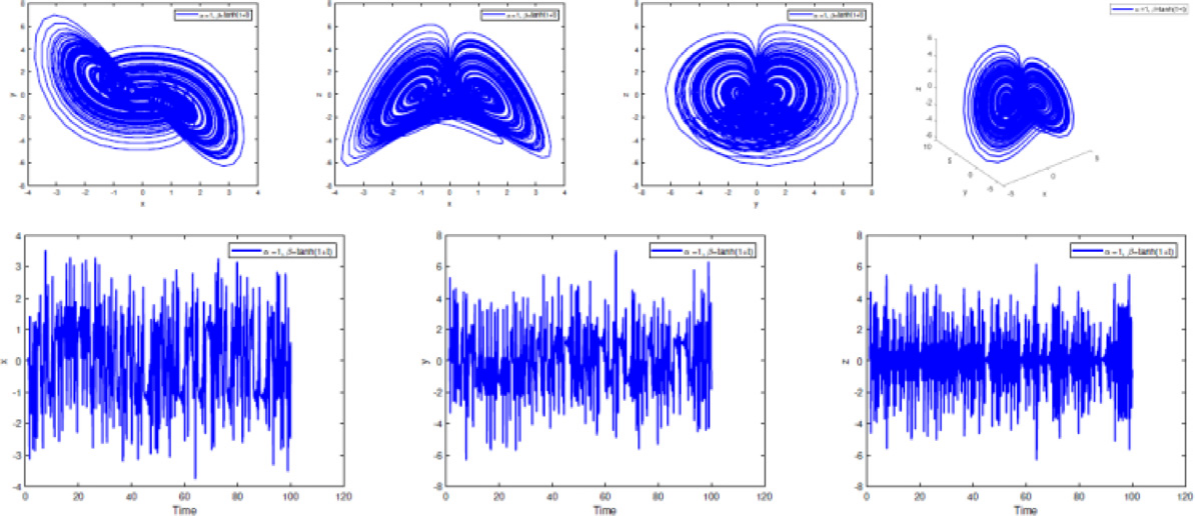
Numerical simulation for fractal fractional Burk-Shaw system using Atangana-Baleanu-Caputo at α=1,β=tanh(1+t).

In Fig. 2, Fig. 3, Fig. 4, Fig. 5, Fig. 6, Fig. 7 is depicted the simulation result (double scroll-attractor) of the fractional-order Burke-Shaw system (1) with parameters a=k=g=10,d=13, orders α=0.95,α=0.97,α=0.99, and initial conditions (x(0),y(0),z(0))=(0.1,0.1,0.1). The x-y-z time series and the phase portraits of the state variables are given in Fig. 2, Fig. 3, Fig. 4, Fig. 5, Fig. 6, Fig. 7. More precisely, 16 are phase trajectories of system (1) projected onto xy,xz,yz,xyz for derivative order β(t)=1,β(t)=0.98,β(t)=0.97+0.03tansh(t/10),β(t)=0.97−0.03sin(t/10),β(t)=0.97−0.03cos(t/10). We can observe that double scroll attractor surrounded the equilibria E2 and E5.

## Discussion

The equilibrium points of system (1) and the corresponding eigenvalues of the Jacobian matrix are shown in Table 1. In the chaotic 3D chaos, the equilibrium points of the Burke-Shaw system (1) that yield all unstable eigenvalue as illustrated in Table 1. A balance with exactly five unstable eigenvalues, the saddle point or saddle focus with index 2, is responsible for the generation of the rolling attractor. It is found in Table 1 that the minimum order corresponding to (1) is amin= 0.94, and chaos may exist above this order. Therefore, the theoretically calculated minimum effective size of Burke-Shaw system is 2.82, and this finding is further verified in the numerical simulation results in Section 4. The system shows better dynamic behavior.

## Conclusion

Fractal-fractional operators could accurately replicate and reveal some chaos. However, because of their non-linearity, their analytical solutions are difficult to obtain and, in some circumstances, impossible to achieve due to their non-linearity. Researchers rely on numerical methods to understand physical behavior. This paper presents a numerical method for chaotic problems. Using fractal-fractional differentiation and integral operators in the sense of Newton interpolation polynomial, we investigated the Burke-Shaw system (1) of mathematical equations able to capture chaotic behavior. Solutions are obtained for the fractional-order Burke-Shaw system (1) using a fractional operator with a non-singular kernel. Uniqueness and boundedness for solution are proved through fixed point theory. Due to the high non-linearity of our problem, we used a suitable numerical scheme to solve this system of equations numerically. The presented scheme is applicable to many other systems, see for example, [10,20,24,25]. For similar numerical results, see [[41], [42], [43], [44], [45], [46], [47], [48], [49], [50], [51]]. In future work, the existence and uniqueness of solutions reported for general component differential equations will be extended to multidimensional problems.

## CRediT authorship contribution statement

**Najat Almutairi:** Conceptualization, Methodology, Software, Data curation, Investigation, Writing – original draft, Writing – review & editing. **Sayed Saber:** Investigation, Writing – review & editing, Supervision, Conceptualization, Methodology, Data curation, Validation.

## Declaration of Competing Interest

The authors declare that they have no known competing financial interests or personal relationships that could have appeared to influence the work reported in this paper.

## Data Availability

No data was used for the research described in the article.
